# An Integrated Approach Combining Chemical Profiling, Network Pharmacology, and Experimental Validation Is Used to Clarify the Pharmacological Basis of the Yiqi‐Tongluo‐Huoxue‐Mingmu Formula in Diabetic Retinopathy

**DOI:** 10.1155/jdr/8484553

**Published:** 2026-05-28

**Authors:** Yuan Gao, Qiang Lyu, Si-wei Wang, Zhu-jun Mao

**Affiliations:** ^1^ School of Pharmaceutical Sciences, Zhejiang Chinese Medical University, Hangzhou, Zhejiang, China, zcmu.edu.cn; ^2^ Panvascular Diseases Research Center, the Quzhou Affiliated Hospital of Wenzhou Medical University, Quzhou People′s Hospital, Quzhou, Zhejiang, China, qzhospital.com

**Keywords:** diabetic retinopathy, LC-Q-TOF/MS, molecular docking, network pharmacology, YQMM

## Abstract

**Background:**

According to traditional Chinese medical (TCM) principles diabetic retinopathy (DR) is categorized as a type of “Wasting‐Thirst Eye Disease.” According to TCM, the pathophysiology of DR is primarily attributed to blood stasis, obstructed collaterals, and concurrent Qi and Yin deficiencies. Yiqi‐Tongluo‐Huoxue‐Mingmu Formula (YQMM), a TCM commonly prescribed for DR, has yet to have its active components and underlying molecular mechanisms fully elucidated.

**Methods:**

The therapeutic efficacy of YQMM was assessed in mice with diabetes induced by streptozotocin (STZ). The chemical profiles of YQMM and its absorbed prototype components in vivo were characterized using LC‐Q‐TOF/MS. After predicting potential targets and pathways using network pharmacology methods and molecular docking, this study validated these computational results through RT‐qPCR experiments. To further evaluate the antiproliferative and antiangiogenic effects of YQMM and its active components, HUVECs cultured under high‐glucose conditions were used.

**Results:**

The retinal protective effects of YQMM were observed without significant alterations in systemic blood glucose levels. Chemical analysis identified 142 compounds, with five prototype constituents (caffeic acid, isoferulic acid, daidzin, daidzein, and mirificin) detected in plasma. Network pharmacology indicated 118 shared targets between YQMM and DR, with key nodes including TP53, ESR1, JUN, STAT3, and MAPK1, primarily linked to the AGE‐RAGE, PI3K‐Akt, and HIF‐1 signaling pathways. Molecular docking predictions revealed strong binding affinities between the targets and the compounds, particularly for daidzin. Both in vivo and in vitro experiments confirmed that YQMM and its active components downregulated the expression of core targets and inhibited high glucose‐induced endothelial cell proliferation and tube formation.

**Conclusion:**

YQMM confers its protective benefits against DR via a complex interplay of multiple active ingredients acting on diverse molecular targets and signaling networks. These findings elucidate the chemical and mechanistic basis for the clinical efficacy of YQMM, supporting its further development as a promising therapeutic agent for DR.

## 1. Introduction

Diabetic retinopathy (DR) is a serious microvascular complication of diabetes and a leading cause of blindness and vision loss worldwide [1]. At early stages of DR, retinal blood vessels become abnormally permeable and some capillaries no longer carry blood. As the disease progresses, these alterations may culminate in pathological neovascularization that threatens vision [2, 3]. Current clinical management of DR primarily relies on intravitreal anti‐VEGF therapy, laser photocoagulation, and strict control of blood glucose and lipid levels. However, these interventions are costly, may cause adverse effects, and often fail to fully restore visual function, underscoring the need for safer and more effective therapeutic strategies [4, 5].

For centuries, traditional Chinese medicine (TCM) has been used to treat diabetes and its complications, and it remains an important source for new therapeutic agents [6]. According to TCM theory, DR is classified as a “Wasting‐Thirst Eye Disease” illness. Blood stasis, collateral blockage, and Qi and Yin deficiencies are the main causes of its pathophysiology. The related therapeutic principle focuses on invigorating blood circulation, eliminating stasis, unblocking collaterals, strengthening Qi, nourishing Yin, and improving vision (summarized as “Huoxue Huayu Tongluo, Fu Zheng Yiqi Yangyin Mingmu”) [7]. Extensive evidence indicates that many Chinese herbal formulas and their active constituents modulate key biological pathways in DR through multitarget and multipathway mechanisms [8–11]. A meta‐analysis of 107 clinical trials involving 9710 patients reported that Chinese herbal therapies significantly improved DR outcomes by lowering blood glucose, reducing retinal hemorrhage, and enhancing visual function, without severe adverse events [12].

The Yiqi‐Tongluo‐Huoxue‐Mingmu Formula (YQMM) is a commonly used clinical prescription formulated under aforementioned therapeutic principle. The formula is composed of six botanicals—namely *Astragalus mongholicus* Bunge. (Huangqi), *Salvia miltiorrhiza* Bunge. (Danshen), *Pueraria montana* var. Lobata (Willd.) Maesen & S.M.Almeida ex Sanjappa & Predeep. (Gegen), *Rhodiola crenulata* (Hook. f. & Thomson) H. Ohba (Hongjing Tian), *Angelica sinensis* (Oliv.) Diels. (Danggui), and *Leonurus japonicus* Houtt. (Chongwei Zi)—each with distinct traditional uses in TCM. Together, these herbs are intended to synergistically tonify Qi and Yin, activate blood circulation, resolve stasis, and unblock collaterals. Despite its clinical use, the pharmacological basis of YQMM remains largely unclear. Most prior studies have concentrated on a few individual herbs (e.g., Huangqi and Gegen) [13, 14], whereas specific investigations of other herbs like Danggui and Hongjing Tian in DR are limited [15, 16]. More importantly, the active chemical constituents of the complete formula and its integrated multitarget mechanism of action remain poorly characterized. The conventional reductionist research model, which focuses on single compounds and single targets, is inadequate for elucidating the synergistic, multipathway effects characteristic of complex TCM formulas like YQMM. Its modernization and a deeper comprehension of its effectiveness are hampered by this information gap. To address these issues, we designed a comprehensive study integrating chemical analysis, computational prediction, and experimental validation. We first used LC‐Q‐TOF/MS to define the chemical profile of YQMM and identify its absorbed prototype compounds in vivo. Subsequently, network pharmacology was employed to predict the candidate molecular targets and signaling pathways through which the formula may exert anti‐DR effects. Finally, key predictions were verified through molecular docking, in vivo pharmacodynamic studies in a diabetic mouse model, and in vitro functional assays using high glucose‐stimulated endothelial cells. This work is aimed at clarifying the material basis and molecular mechanisms of YQMM, providing a scientific rationale for its clinical use in DR treatment.

Traditional research frameworks centered on a single drug acting on a single target are inadequate for TCM prescriptions, whose clinical efficacy generally arises from complex synergistic effects produced by multiple ingredients interacting with diverse biological targets and regulatory networks. This paradigm is particularly limited in elucidating the integrative mechanisms underlying formulas like YQMM, creating a significant bottleneck that hampers both mechanistic understanding and clinical translation. Network pharmacology offers a powerful, systems‐level alternative. By constructing “compound‐target‐disease” interaction networks, it enables the high‐throughput prediction and visualization of regulation patterns from a holistic perspective. This strategy represents a paradigm shift in TCM research, moving away from a narrow focus on isolated drug‐target interactions toward a holistic model in which multiple constituents act coordinately across networks of targets and biological pathways [17]. Network topology can uncover the combinatorial models and functional characteristics of herbal formulae, providing an important foundation for exploring their underlying mechanisms [17, 18].

Consequently, in contrast to earlier approaches that concentrated on individual herbs or single signaling pathways, this study adopted a comprehensive strategy integrating chemical analysis, computational prediction, and experimental validation in order to overcome current research bottlenecks and systematically elucidate the pharmacologically active components and synergistic mechanisms of the YQMM. In particular, we constructed an interaction network between YQMM and DR by integrating LC‐Q‐TOF/MS‐based component identification with network‐pharmacology inference and experimental validation. This study provided direct experimental proof for the pharmacological foundation of YQMM by methodically identifying its five in vivo archetypal components and in vitro chemical constituents for the first time. Additionally, it demonstrated for the first time that these components can synergistically regulate the AGE‐RAGE/PI3K‐Akt/HIF‐1 signaling network without depending on hypoglycemic effects by concurrently acting on multiple core nodes, such as TP53, STAT3, JUN, and other core nodes, rather than a single target.

## 2. Materials and Methods

### 2.1. Extraction of YQMM

The Chinese traditional medicine was purchased from Huqingyutang (Hangzhou, Zhejiang, China). First, the previously indicated herbs were weighed in a ratio of and steeped for 2–3 h in filtered water at a 1:10 (*w*/*v*) ratio. Subsequently, the solution was then boiled for 40 min, followed by maintaining a gentle boil for 30 min and filtered with gauze. The decoction was obtained by filtration. Collect the filter residue, add eight times its volume of water, and continue heating to extract once more. Finally, the filtrates obtained from the two extractions were combined and concentrated using a rotary evaporator to a density of 1 g/mL (equivalent to 1 g of crude drug per milliliter).

### 2.2. Animal

In this study, the aged 6–8 weeks and weighing between 18 g and 20 g male mice (C57BL/6 J) were obtained. The animals were housed in a specific pathogen free (SPF) facility and cared for under controlled conditions throughout the study. After 7 days of acclimatization, the mice were maintained on a light/dark cycle, with ambient temperatures controlled between 22°C–26°C and relative humidity set at 50%–60%. Throughout the study, they had free access to standard chow and drinking water. All procedures involving animals were reviewed and approved by the Animal Ethics Committee of Zhejiang Chinese Medical University (IACUC‐20231009‐11).

### 2.3. Model Establishment and Drug Administration

Seven‐week‐old mice were first divided into two groups at random: the model group and the control group (*n* = 10). To induce diabetes, mice were fasted for 2 h with free access to water. Subsequently, a freshly prepared STZ solution (55 mg/kg, sodium citrate buffer, pH 4.5) was administered via intraperitoneal injection. The experimental procedure lasted for 5 days. One week after the final injection, fasting blood glucose was monitored for three successive days. Mice that consistently exhibited fasting glucose readings above 13.9 mmol/L during this period were confirmed as diabetic and selected for further study.

Successfully induced animals were randomly assigned to five experimental groups: the STZ group (*n* = 15), the YQMM‐L (*n* = 13), YQMM‐M (*n* = 13), YQMM‐H (*n* = 13) group, and the insulin group (*n* = 13), which served as a reference control. The following were the dosages: YQMM‐L: 100 mg/kg; YQMM‐M: 200 mg/kg; YQMM‐H: 400 mg/kg; Insulin: 4 U/kg/day. YQMM was delivered by oral gavage, whereas insulin was injected subcutaneously every day for 10 weeks. Blood glucose levels and body weight were measured every 7 days during the course of the medication.

### 2.4. Fluorescein Fundus Angiography (FFA)

FFA was used to evaluate fundus retinal vascular lesions. After anesthetizing the mouse, the pupils were fully dilated by administering 0.5% tropicamide (Santen, Japan). Subsequently, 10 *μ*L/kg body weight of 1% sodium fluorescein solution (Alcon Laboratories, Inc., United States) was injected into the tail vein. Use the Confocal Laser Fundus Camera CRO fundus contrast imaging equipment to take pictures right after injection. Researchers unaware of the grouping performed analysis of vascular branching points using AngioTool software (NIH, Bethesda, Maryland, United States) [19].

### 2.5. Periodic Acid‐Schiff Stain (PAS)

Following established protocols [20], PAS‐based histochemical labeling enabled enumeration of capillary ghosts in the retina. Briefly, the whole eyeballs were carefully treated to ensure thorough fixation. After dissecting out the retina, the tissue was incubated in a 3% trypsin enzyme mixture for about 2 h to digest away nonvascular elements. The isolated vascular networks were then visualized by staining with a PAS reagent kit following the supplier′s protocol.

### 2.6. YQMM Component Analysis

#### 2.6.1. In Vitro and In Vivo Sample Preparation

The YQMM solution was freeze‐dried for 48 h before the experiment. A sample solution with a mass concentration of 50 mg/mL was created by redissolving the resultant powder in 70% methanol. The supernatant was collected for analysis following a 10 min centrifugation at 4°C. After random allocation into either a YQMM‐treated group or an untreated control group, C57BL/6 mice received daily oral dosing over a 3‐day span. Thirty minutes after the final treatment, samples of blood were obtained from the retro‐orbital venous plexus for subsequent testing, 1 h, and 2 h into prechilled anticoagulant tubes for subsequent analysis. After that, the samples were centrifuged to extract the supernatant, or plasma. Equal volume mixing was then used to pretreat plasma samples from both groups at each time point. To precipitate proteins, mixed plasma samples were combined with five times as much mass spectrometry‐grade methanol. After vortex agitation and ultrasonic treatment, the mixture was centrifuged for 12 h at 4°C overnight. After being collected and vacuum‐dried for 48 h, the supernatant was resuspended in methanol with an internal standard. The final supernatant was collected for analytical testing following centrifugation.

#### 2.6.2. LC‐Q‐TOF/MS

LC‐Q‐TOF/MS analysis was carried out using a Waters Acquity UPLC I‐class system coupled with a Xevo G2‐XS QTOF mass spectrometer. The chromatographic separation was performed on an ACQUITY UPLC HSS T3 column (100 × 2.1 mm, 1.8 *μ*m) as the stationary phase. The mobile phase consisted of solvent A (0.1% formic acid in water) and solvent B (0.1% formic acid in acetonitrile). A gradient elution was employed as outlined in Table 1.

**Table 1:** LC‐Q‐TOF/MS gradient elution program. Phase A: 0.1% formic acid in water, *v*/*v*; Phase B: 0.1% formic acid in acetonitrile, *v*/*v*.

Time (min)	Phase A (%)	Phase B (%)
0	98	2
2	95	5
10	85	15
15	75	25
18	50	50
23	0	100
25	98	2
30	98	2

Mass analysis was carried out using an electrospray source capable of generating both positively and negatively charged ions prior to detection ([*M* + *H*]^+^and [M−H]^−^) was used. LockSpray technology was employed to ensure data accuracy. The ESI source was maintained at 100°C with a desolvation temperature of 500°C. Instrument settings were optimized so that the ion source operated at a high voltage, with auxiliary gas flows tuned for efficient desolvation and ion transfer prior to detection. The system was run in a mode that automatically selects precursor ions for fragmentation based on signal intensity. Both survey and product ion spectra were acquired over a broad *m*/*z* range with rapid duty cycles, allowing multiple of the most abundant features in each cycle to be targeted for structural interrogation. Precursors were excluded from repeated selection for a short interval to maximize coverage, and collision energy was varied across the mass range to ensure effective breakdown of ions into informative fragments. All measurements were recorded in continuous signal mode, with stringent mass precision tolerances maintained throughout the acquisition.

#### 2.6.3. Data Processing and Analysis

Using Mass Lynx V4.2 software, we employ a hierarchical qualitative strategy by referencing mass spectrometry data from public databases such as GNPS, literature sources, PubChem, and Massbank. This involves manually comparing secondary spectra from YQMM with those in the databases to ensure accuracy, thereby enabling compound identification. The analytical workflow employed is as follows: raw data for YQMM and blood compounds obtained via LC‐Q‐TOF/MS acquisition are converted into mzML files using MS convert (https://protogwizard.sourceforge.io), then uploaded to GNPS (http://gnps.ucsd.edu) for analysis via WinSCP.

### 2.7. Target Prediction

To forecast possible protein targets, the Swiss Target Prediction database received chemical structures (SMILES for “mat”) of the prototype chemicals absorbed into blood (identified in section 2.6). All targets with a likelihood score greater than zero were kept, and predictions were limited to *Homo sapiens*. In parallel, compound‐associated protein targets that had been experimentally validated were retrieved from the PubChem repository. To ensure uniformity in gene nomenclature, all target identifiers were mapped to their corresponding official gene symbols by referencing the UniProt knowledgebase. To create a distinct list of possible YQMM targets, duplicates were eliminated. Four public databases were used to systematically extract disease‐associated targets for “diabetic retinopathy”. Targets in GeneCards were chosen if their relevance score was higher than the average. A complete set of DR‐related targets was created by combining targets from all sources, eliminating duplicates, and standardizing gene names using UniProt. The common targets, which were thought to be possible therapeutic targets for YQMM in DR treatment, were obtained by intersecting the potential YQMM targets with the DR‐related targets. The MicroBioinformatics web platform (https://www.bioinformatics.com.cn/) was used to create a graphic Venn diagram. A “Compound‐Target‐Disease” network was created and examined using Cytoscape software (Version 3.10.3) in order to show the intricate relationships. Compounds, targets, or the illnesses were represented by nodes in this network, whereas interactions were represented by edges.

### 2.8. Building the Interactome and Isolating Principal Network Nodes

Based on the common targets found in the preceding section, important proteins (hub proteins) at the core of interaction networks are identified to provide a foundation for subsequent mechanism studies. First, input the aforementioned intersection targets into the STRING database′s Multiple proteins search feature (https://string-db.org/, Version 12.0). Create the YQMM disease‐target protein–protein interaction (PPI) network, and hid the disconnected nodes, as well as leave the default parameters. Export the outcomes as a file in TSV format. Subsequently, visualize the downloaded TSV file using Cytosccape software. Target screening was conducted using literature reports and the CytoNCA plugin. The CytoNCA plugin identified nodes with median, proximity, and connectivity values above the median as key network nodes.

### 2.9. GO‐Based Functional Characterization and KEGG Route Exploration

In order to further investigate how YQMM may influence DR at a pathway level, the intersecting candidate list was annotated using GO terms and KEGG maps. Common genes were submitted to the DAVID bioinformatics resource for pathway enrichment studies, using official human gene symbols as input identifiers. GO enrichment output was summarized across the three ontology domains. Only enrichment terms meeting statistical criteria (e.g., FDR or *p* value < 0.05) were retained. From the filtered results, the Top 10 most significant terms in each GO category and the Top 20 enriched KEGG pathways were selected for further interpretation. To visually summarize these findings, bar plots and bubble charts were generated through the MicroBioinformatics platform. For information on constructing the “component‐target‐pathway” network, please refer to the Supporting Information.

### 2.10. Molecular Docking

In this study, the molecular docking technique was used to predict receptor–ligand interactions, determine binding orientations, and estimate binding affinities [21]. Retrieve the protein receptor ID from the UniProt database. Enter the ID into the PDB database for searching and download the PDB format file. Use PyMOL to remove solvents and ligands from the protein receptor. To eliminate ligands and solvents from the protein receptor, use PyMOL. Next, use Chem3D software to transform the small‐molecule ligand′s 2D structure (SDF format) from the PubChem database into a 3D structure (MOL2 format). After performing hydrogenation, charge assignment, and protonation optimization on these files using Autodocktools 1.5.7, they are saved as PDBQT format files. Lastly, use Autodocktools 1.5.7 and the Autodockvina program to carry out molecular docking. Typically, the more stable the conformation of a small molecule bound to a protein, the lower its energy, the greater the probability of interaction occurring, and the more reliable the docking results.

### 2.11. Cell Culture and Drug Treatment

Human umbilical vein endothelial cells (HUVECs, Zhongqiao Xinzhou, China) were cultured at 37°C in a humidified atmosphere with 5% CO_2_ in endothelial cell growth medium. The cells were subcultured every 2–3 days using 0.25% trypsin‐EDTA, and experiments were performed on cells between passages 3 and 6. The following treatment conditions were applied as follows: (1) LG: 5.5 mM D‐glucose; (2) HG: 30 mM D‐glucose; (3) CA‐L: 1 *μ*M; CA‐H: 10 *μ*M; (4) IFA‐L: 5 *μ*M; IFA‐H: 50 *μ*M; (5) mirificin‐L: 10 *μ*M; mirificin‐H: 50 *μ*M; (6) daidzin‐L: 5 *μ*M; daidzin‐H: 50 *μ*M; (7) daidzein‐L: 5 *μ*M; daidzein‐H: 10 *μ*M; (8) YQMM‐L: 1 *μ*g/mL; YQMM‐L: 10 *μ*g/mL. Mannitol was added to the normal glucose medium to adjust for osmotic pressure. Treatments were administered for 24 h prior to analysis. CA, IFA, mirificin, daidzin, and daidzein were purchased from ChemFaces (Wuhan, China).

### 2.12. Tube Formation Assays and EDU

To investigate whether the bioactive constituents of YQMM ameliorate high glucose‐induced endothelial dysfunction. In this study, a Matrigel‐based tube formation assay was performed, and quantitative analysis was conducted via digital image analysis using ImageJ software. Proliferative activity was evaluated by measuring EdU incorporation using a click‐chemistry fluorescence kit in accordance with the vendor′s protocol.

### 2.13. RT–qPCR–Based Transcript Measurement

RNA was prepared from each specimen with TransZol Up (TransGen, Beijing, China; ET111‐01‐V2). First‐strand cDNA was synthesized using PrimeScript RT Master Mix. Real‐time amplification was performed with a SYBR‐based mix (Aikrui, Hunan, China; AG11701) on a qPCR instrument. Relative mRNA abundance was reported as fold change using the 2^−*ΔΔ*Ct^ approach. Primer oligonucleotides were commercially produced and are provided in Table 2.

**Table 2:** The primers used in our current study.

Primer name	Forward (5 ^′^−3 ^′^)	Reverse (5 ^′^−3 ^′^)
m‐Tp53	GCGTAAACGCTTCGAGATGTT	TTTTTATGGCGGGAAGTAGACTG
H‐TP53	GAGGTTGGCTCTGACTGTACC	TCCGTCCCAGTAGATTACCAC
m‐STAT3	CACCTTGGATTGAGAGTCAAGAC	AGGAATCGGCTATATTGCTGGT
H‐STAT3	ACCAGCAGTATAGCCGCTTC	GCCACAATCCGGGCAATCT
m‐MAPK1	GGTTGTTCCCAAATGCTGACT	CAACTTCAATCCTCTTGTGAGGG
H‐MAPK1	TACACCAACCTCTCGTACATCG	CATGTCTGAAGCGCAGTAAGATT
m‐Jun	ACTCGGACCTTCTCACGTC	GGTCGGTGTAGTGGTGATGT
H‐JUN	TCCAAGTGCCGAAAAAGGAAG	CGAGTTCTGAGCTTTCAAGGT
m‐Esr1	TGTGTCCAGCTACAAACCAATG	CATCATGCCCACTTCGTAACA
m‐Gapdh	TGTGTCCGTCGTGGATCTGA	TTGCTGTTGAAGTCGCAGGAG
H‐*β*‐actin	CCTGGACTTCGAGCAAGAGATGG	CAGGAAGGAAGGCTGGAAGAGTG

### 2.14. Statistical Analysis

Until the preliminary analysis of the experimental data is finished, all information about drug treatments and group assignments will be kept private by the staff members in charge of FFA image analysis, PAS staining counts, and qPCR operations.

All statistical computations were conducted in GraphPad Prism Version 9.5. Data are presented as mean ± standard deviation. To examine differences among groups, appropriate statistical tests were selected based on data characteristics. Normality of distribution was first evaluated using the Shapiro–Wilk test. For variables meeting normal distribution assumptions, differences between two groups were assessed using Student′s *t*‐test, whereas multigroup comparisons were analyzed by two‐way analysis of variance. For nonparametric data, comparisons were made using the Kruskal–Wallis test or the Wilcoxon signed‐rank test as appropriate. A two‐sided *p* value less than 0.05 was considered to indicate statistical significance.

## 3. Results

### 3.1. YQMM Ameliorates Retinal Vascular Pathology in DR Mice

**Figure 1:**
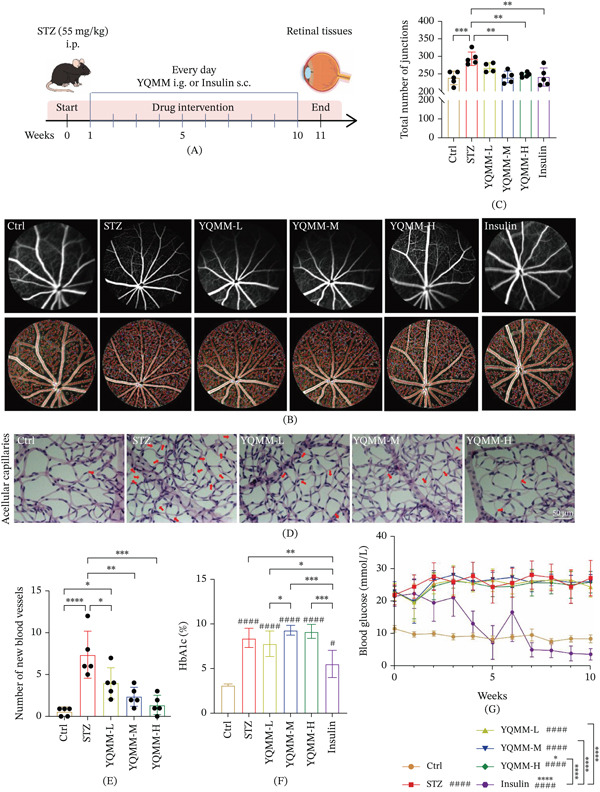
Pharmacodynamic study of YQMM on DR mice. (A) Schematic diagram of the in vivo experimental workflow for YQMM. (B) FFA images of mice treated according to specified protocols. (C) Analysis using AngioTool software to count total vascular branch points. Data presented as mean ± standard deviation (*n* = 4–5). (D–E) Representative micrographs of retinal trypsin digests (PAS‐stained) and the quantification of acellular capillaries in mice (magnification: 40×; scale bar = 50 *μ*m; *n* = 5). (F–G) Glycated hemoglobin and fasting blood glucose levels in mice. #: Compared with ctrl group, ^####^ *p* < 0.0001, ^###^ *p* < 0.001, ^##^ *p* < 0.01, ^#^ *p* < 0.05;  ^∗^: Compared with STZ group,  ^∗∗∗∗^ *p* < 0.0001,  ^∗∗∗^ *p* < 0.001,  ^∗∗^ *p* < 0.01,  ^∗^ *p* < 0.05.

To evaluate the therapeutic effect of YQMM on DR, a streptozotocin‐induced Type 1 diabetic mouse model was established (Figure 1A). According to FFA study (Figure 1B,C), retinal vascular branching points were significantly higher in diabetes mice than in the nondiabetic control group. Compared with untreated diabetic mice, YQMM administration markedly reduced the excessive formation of aberrant vascular branches.

Consistent with the FFA findings, analysis of retinal trypsin digests with PAS staining indicated that sustained hyperglycemia significantly increased the number of acellular capillaries, a hallmark of DR vasculopathy (Figure 1D,E). Administration of YQMM effectively attenuated this damage, decreasing both the density of acellular capillaries and the occurrence of aberrant, tortuous vessels (indicated by red arrows in Figure 1D). These findings show that YQMM improves aberrant vascular morphology and reduces vascular proliferation in the DR.

As the positive control group, insulin therapy significantly reduced the number of acellular capillaries and the number of retinal vascular branch sites. This effect may be attributable to the pronounced systemic glucose‐lowering action of insulin. In contrast, YQMM did not significantly change blood glucose or glycated hemoglobin levels in diabetic mice, despite effectively improving retinal vascular pathology (Figure 1F,G). These findings suggest that the protective effect of YQMM against DR may be mediated through mechanisms other than systemic glucose lowering.

### 3.2. Comprehensive Chemical Profiling and Identification of Systemically Absorbed Constituents of YQMM

**Figure 2:**
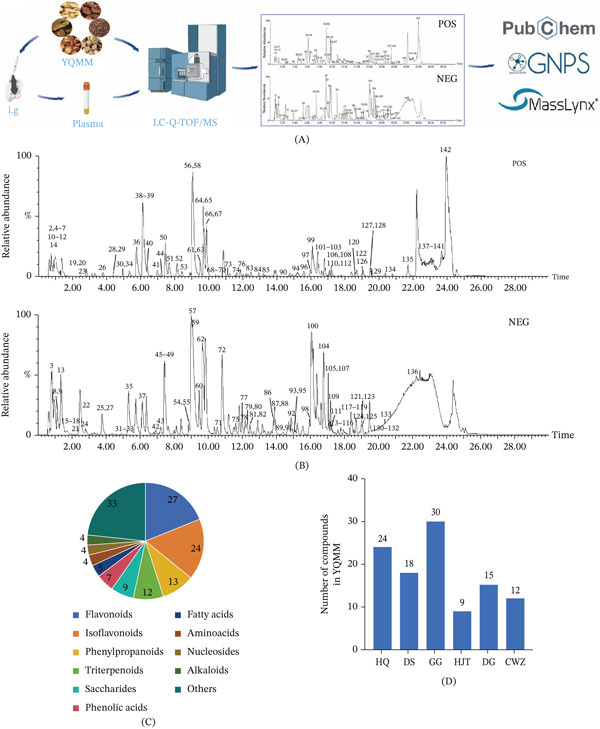
Chemical identification of YQMM was performed using LC‐Q‐TOF/MS. (A) Workflow of component analysis. (B) Base peak ion (BPI) chromatograms of YQMM in positive and negative ion modes. (C) Structural classification of compounds identified in YQMM. (D) Number of identified chemical constituents in each medicinal herb. HQ: HuangQi; DS: DanShen; GG: GeGen; HJT: HongJingtian; DG: DangGui; CWZ: ChongWeizi.

LC‐Q‐TOF/MS was used in an untargeted analysis to describe the chemical composition of YQMM (Figure 2A). Representative base peak intensity (BPI) chromatograms acquired in both ion modes are shown in Figure 2B. The acquired MS/MS data were processed by spectral matching against the GNPS database, and putative identifications were further verified by manually comparing fragmentation patterns with reference spectra from public databases (e.g., PubChem and MassBank) and the relevant literature using MassLynx software (v4.2).

Figure 2C summarizes the identification of 142 chemicals from the YQMM extract. Each herbal component was assigned the following number of compounds: Danshen (DS, 18), Gegen (GG, 30), Hongjing Tian (HJT, 9), Danggui (DG, 15), Chongwei Zi (CWZ, 12), and Huangqi (HQ, 24) (Figure 2D). Interestingly, a number of chemicals were found in many herbs; for instance, CA was found in Huangqi, Danshen, and Hongjing Tian.

Representative compounds identified in the formula included salvianolic acid A, a major bioactive constituent of Danshen [22]; puerarin, an isoflavone from Gegen; and CA, which was detected in Huangqi, Danshen, and Hongjing Tian. These compounds have demonstrated multifaceted pharmacological potential in studies of diabetes and its complications. The observed protective effects are likely mediated by a combination of anti‐inflammatory, antioxidant, anti‐VEGF, antiapoptotic, and hypoglycemic actions, collectively contributing to the attenuation of retinal vascular pathology, including suppression of aberrant neovascularization [14, 23–25]. A detailed summary of each compound detected in our analysis is presented in Table 3, which includes parameters such as retention time, molecular formula, exact mass, and corresponding MS/MS fragmentation profiles.

**Table 3:** Identification of the chemical constituents of YQMM.

NO.	RT (min)	Formula	Name	Adduct	MS	MS/MS	Category	Source
1	0.291	C_10_H_30_O_5_Si_5_	Cyclomethicone 5	[M + H]^+^	371.1027	355.06, 266.99, 73.05	/	CWZ
2	0.741	C_6_H_14_N_4_O_2_	Arginine	[M + H]^+^	175.1199	175.12, 130.10, 116.07, 70.07, 60.06	Amino acids	DG
3	0.859	C_24_H_42_O_21_	Stachyose	[M−H]^−^	665.2152	383.12, 179.06, 119.03, 89.03, 89.03	Saccharides	HQ, DG
4	0.900	C_18_H_32_O_16_	Maltotriose	[M + H‐H_2_O]^+^	488.2857	325.11, 163.06, 145.05, 85.03	Saccharides	HQ
5	0.904	C_18_H_32_O_16_	Raffinose	[M + H]^+^	505.2543	325.11, 163.06, 85.03	Saccharides	GG
6	0.914	C_12_H_22_O_11_	Sucrose	[M + H‐H_2_O]^+^	325.1330	145.05, 127.04, 97.02, 85.03, 69.04	Saccharides	DG
7	1.020	C_12_H_22_O_11_	Melibiose	[M + Na]^+^	365.1064	360.25, 163.02, 145.02, 127.77, 103.31	Saccharides	HJT
8	1.095	C_6_H_8_O_7_	Citric acid	[M−H]^−^	191.0175	111.01, 87.01, 85.03, 67.02	Fatty acids	GG, CWZ, HJT
9	1.122	C_24_H_42_O_21_	2‐[[6‐[[6‐[3,4‐Dihydroxy‐2,5‐bis(hydroxymethyl)oxolan‐2‐yl]oxy‐3,4,5‐trihydroxyoxan‐2‐yl]methoxy]‐3,4,5‐trihydroxyoxan‐2‐yl]methoxy]‐6‐(hydroxymethyl)oxane‐3,4,5‐triol	[M + HCOO]^−^	711.2206	666.27, 667.22, 665.21, 101.02	Saccharides	/
10	1.173	C_24_H_42_O_21_	Stachyose hydrate	[M + Na]^+^	689.2119	689.21, 527.16	Saccharides	CWZ
11	1.215	C_18_H_32_O_16_	Melezitose	[M + Na]^+^	527.1572	365.10, 347.09, 203.05, 145.06	Saccharides	/
12	1.325	C_9_H_11_NO_3_	Tyrosine	[M + H]^+^	182.0802	136.07, 123.05, 119.05, 95.06, 91.06	Amino acids	DG
13	1.343	C_11_H_17_NO_8_	N‐fructosyl pyroglutamate	[M−H]^−^	290.0868	290.09, 212.05, 201.05, 200.06, 128.03	/	DG
14	1.401	C_6_H_14_N_4_O_2_	Arginine	[M + H−NH_3_]^+^	158.0169	159.03, 158.02, 117.09, 115.09	Amino acids	DG
15	1.678	C_9_H_12_N_2_O_6_	Uridine	[M−H]^−^	243.0662	243.07, 200.06, 111.01, 110.0241	Nucleosides	HQ, DG
16	1.770	C_14_H_12_O_4_	Piceatannol	[M−H]^−^	243.0636	243.06, 201.12, 199.07	/	DG, DS
17	1.908	C_5_H_8_O_5_	Citramalic acid	[M−H]^−^	147.0291	147.03, 129.02, 103.04, 87.01, 85.03	Fatty acids	HQ
18	2.022	C_12_H_16_O_7_	4‐Hydroxyphenyl hexopyranoside	[M + HCOO]^−^	317.0784	317.08, 271.09, 163.04, 161.06, 108.02	/	CWZ
19	2.136	C_12_H_23_NO_7_	N‐fructosyl isoleucine	[M + H]^+^	294.1595	294.16, 276.15, 258.13, 230.13, 212.12	/	/
20	2.290	C_10_H_13_N_5_O_5_	Guanosine	[M + H]^+^	284.0935	153.07, 152.05, 135.04, 110.03	Nucleosides	CWZ
21	2.296	C_10_H_13_N_5_O_5_	Arabinosylguanine	[M−H]^−^	282.0847	282.08, 150.04, 133.01, 108.02, 107.04	Nucleosides	GG
22	2.639	C_7_H_6_O_5_	Gallic acid	[M−H]^−^	169.0137	169.01, 168.79, 127.03, 125.02, 97.55	Phenolic acids	GG
23	2.711	C_10_H_20_N_4_O_3_	2‐(Butanoylamino)‐5‐(diaminomethylideneamino)pentanoic acid	[M + H]^+^	245.1620	245.16, 228.14, 186.11, 175.12, 158.09, 116.07	/	/
24	2.802	C_20_H_20_O_14_	1,6‐Digalloyl‐beta‐D‐glucopyranose	[M−H]^−^	483.0766	483.08, 313.06, 169.01, 151.01, 125.02	Phenolic acids	DS
25	3.749	C_9_H_8_O_4_	Caffeic acid	[M−H]^−^	179.0350	179.04, 135.04, 107.05, 91.05	Phenylpropanoids	HQ, DS, HJT
26	3.772	C_8_H_8_O_3_	3‐Methylsalicylic acid	[M + H]^+^	153.0429	153.04, 125.02, 111.05, 93.08	Phenolic acids	/
27	3.780	C_9_H_10_O_5_	Salvianic acid A	[2 M−H]^−^	395.0984	197.05, 179.03, 135.04	Phenolic acids	DS
28	4.318	C_14_H_17_N_5_O_8_	Succinoadenosine	[M + H]^+^	384.1145	252.07, 192.06, 162.08, 136.06	Nucleosides	/
29	4.519	C_17_H_24_O_12_	3‐[(2S,3R,4S,5S,6R)‐6‐[[(2R,3R,4R)‐3,4‐dihydroxy‐4‐(hydroxymethyl)oxolan‐2‐yl]oxymethyl]‐3,4,5‐trihydroxyoxan‐2‐yl]oxy‐2‐methylpyran‐4‐one	[M + Na]^+^	443.1193	443.12, 317.08	/	/
30	4.968	C_11_H_12_N_2_O_2_	Tryptophan	[M + H]+	205.0956	188.07, 159.09, 146.06, 144.08, 118.07	Amino acids	CWZ
31	5.079	C_16_H_18_O_9_	Caffeoylquinic acid	[M−H]^−^	353.0852	355.17, 354.09, 353.09, 191.06, 179.03	Phenylpropanoids	CWZ
32	5.117	C_16_H_18_O_9_	New chlorogenic acid	[M−H]^−^	353.0936	191.06, 84.28, 58.87	Phenylpropanoids	CWZ
33	5.124	C_15_H_20_O_10_	2MeO	[M−H]^−^	359.0298	198.05, 197.05, 182.02, 153.06, 153.05	/	HQ, GG
34	5.156	C_16_H_18_O_9_	Chlorogenic acid	[M + Na]^+^	377.0927	355.91, 163.04	Phenylpropanoids	CWZ
35	5.223	C_9_H_10_O_4_	2‐Hydroxy‐3‐(4‐hydroxyphenyl)propanoic acid	[M‐H]^-^	181.0509	181.05, 164.05, 163.03, 135.04, 119.05	/	/
36	5.825	C_8_H_11_NO	Tyramine	[M + H‐NH_3_]^+^	121.0644	121.06, 103.05, 93.07, 91.05	/	/
37	6.068	C_27_H_30_O_15_	Isovitexin‐7‐O‐glucoside	[M−H]^−^	593.1506	503.12, 473.10, 311.05, 282.05	Flavonoids	CWZ
38	6.081	C_15_H_10_O_5_	7,3 ^′^,4 ^′^‐Trihydroxyisoflavone	[M + H]^+^	271.0402	271.12, 197.96	Isoflavonoids	HQ, GG, DG
39	6.111	C_28_H_32_O_8_	NCGC00385480‐01	[M + Na]^+^	519.2378	520.22, 519.24, 272.12, 271.12	/	/
40	6.431	C_14_H_18_N_2_O_2_	Lenticin	[M + H]^+^	247.1403	189.08, 188.07, 146.08, 144.08, 118.07, 60.08	Alkaloids	GG
41	6.907	C_28_H_32_O_15_	Spinosin	[M + H]^+^	609.1963	609.20, 447.13, 429.13, 381.10, 351.08, 327.08	Flavonoids	CWZ
42	6.911	C_15_H_14_O_6_	Catechin	[M−H]^−^	289.0726	289.07, 245.08, 125.02, 123.05, 109.03	Flavonoids	HJT
43	7.119	C_16_H_28_O_10_	3‐Methyl‐2‐butenyl 6‐O‐alpha‐L‐arabinopyranosyl‐beta‐D‐glucopyranoside	[M + HCOO]^−^	425.1664	425.17, 380.16, 379.16, 149.04, 119.77	/	DG
44	7.21	C_16_H_28_O_10_	2‐Methyl‐3‐buten‐2‐yl 6‐O‐[(2R,3R,4R)‐3,4‐dihydroxy‐4‐(hydroxymethyl)tetrahydro‐2‐furanyl]‐beta‐D‐glucopyranoside	[M + Na]^+^	403.1579	404.16, 403.16, 335.09, 333.08	/	HJT
45	7.405	C_21_H_20_O_10_	3‐Genistein‐8‐C‐glucoside	[M−H]^−^	431.0976	311.06, 283.06	Isoflavonoids	GG
46	7.408	C_21_H_20_O_10_	5,7‐Dihydroxy‐2‐(4‐hydroxyphenyl)‐8‐[3,4,5‐trihydroxy‐6‐(hydroxymethyl)oxan‐2‐yl]chromen‐4‐one	[2 M−H]^−^	863.3955	431.10, 341.07, 312.05, 311.06, 283.06	Flavonoids	/
47	7.457	C_21_H_20_O_10_	Apigenin‐7‐O‐glucoside	[M−H]^−^	431.0965	433.10, 432.10, 431.10, 269.04, 268.03	Flavonoids	HQ
48	7.457	C_21_H_20_O_10_	Kaempferol‐3‐O‐rhamnoside	[M−H]^−^	431.0975	432.10, 431.10, 286.07, 285.05, 284.03	Flavonoids	CWZ
49	7.462	C_21_H_20_O_10_	Emodin‐8‐glucoside	[2 M−2H + Na]^2−^	885.1890	431.10, 413.09, 311.06, 269.96	/	DS
50	7.523	C_21_H_20_O_10_	Vitexin	[M + H]^+^	433.1139	415.10, 397.09, 367.08, 313.07, 283.06	Flavonoids	DS
51	7.700	C_21_H_20_O_10_	Genistin	[M + H]^+^	433.1095	433.11, 271.12, 271.06	Isoflavonoids	GG
52	8.019	C_21_H_20_O_11_	Isoorientin	[M + H]^+^	449.1017	451.11, 450.18, 449.11, 431.19	Flavonoids	GG, DS
53	8.412	C_26_H_28_O_14_	8‐[3,5‐Dihydroxy‐6‐(hydroxymethyl)‐4‐(3,4,5‐trihydroxyoxan‐2‐yl)oxyoxan‐2‐yl]‐5,7‐dihydroxy‐3‐(4‐hydroxyphenyl)chromen‐4‐one‐hydroxyphenyl)chromen‐4‐one	[M + H]^+^	565.157	565.16, 433.11, 415.10, 313.07, 283.05	Isoflavonoids	/
54	8.924	C_27_H_32_O_15_	2,6‐dihydroxy‐2‐[(4‐hydroxyphenyl)methyl]‐4‐[(2S,3R,4S,5S,6R)‐3,4,5‐trihydroxy‐6‐[[(2R,3R,4R,5R,6S)‐3,4,5‐trihydroxy‐6‐methyloxan‐2‐yl]oxymethyl]oxan‐2‐yl]oxy‐1‐benzofuran‐3‐one	[M−H]^−^	595.1672	259.06, 178.99, 125.02	Flavonoids	/
55	8.931	C_15_H_10_O_5_	3 ^′^‐Hydroxydaidzein	[M−H]^−^	269.0457	239.03, 223.03, 211.04, 135.01, 119.01	Isoflavonoids	GG
56	9.055	C_21_H_20_O_9_	Daidzin	[M + H]^+^	417.1187	833.24, 419.12, 418.12, 417.12, 255.08	Isoflavonoids	GG
57	9.114	C_21_H_20_O_9_	7‐hydroxy‐3‐(4‐hydroxyphenyl)‐8‐[3,4,5‐trihydroxy‐6‐(hydroxymethyl)(2H‐3,4,5,6 ‐tetrahydropyran‐2‐yl)]chromen‐4‐one	[M−H]^−^	415.1033	415.10, 296.06, 295.06, 267.05, 268.07	Isoflavonoids	/
58	9.151	C_21_H_20_O_9_	Puerarin	[M + H]^+^	417.1186	417.12, 399.11, 381.10, 297.08, 267.07	Isoflavonoids	GG
59	9.210	C_21_H_20_O_9_	Daidzin—20 eV	[M−H]^−^	415.1095	416.11, 415.11, 254.05, 253.05, 252.04	Isoflavonoids	GG
60	9.421	C_21_H_20_O_11_	Luteolin‐8‐C‐glucoside	[M−H]^−^	447.0928	447.09, 357.06, 328.06, 327.05	Flavonoids	HJT
61	9.523	C_17_H_20_N_4_O_6_	Riboflavine	[M + H]^+^	377.1475	377.15, 243.09, 198.07, 172.08	Alkaloids	HQ
62	9.645	C_22_H_22_O_10_	6‐beta‐D‐Glucopyranosyl‐4 ^′^,5‐dihydroxy‐7‐methoxyflavone	[2 M−H]^−^	891.0952	445.11, 325.07, 297.07, 297.08, 282.05	Flavonoids	DS
63	9.676	C_22_H_22_O_10_	Glycitein 7‐O‐glucoside	[M + H]^+^	447.1286	915.21, 469.11, 447.13, 285.08	Isoflavonoids	GG
64	9.727	C_22_H_22_O_10_	Sissotrin	[M + H]^+^	447.1291	447.13, 286.06, 285.08, 270.08, 152.00	Isoflavonoids	GG
65	9.770	C_22_H_22_O_10_	3 ^′^‐MethoxyPuerarin	[M + H]^+^	447.1290	429.12, 327.09, 297.08	Isoflavonoids	GG
66	9.856	C_26_H_28_O_13_	Mirificin	[M + H]^+^	549.1602	549.16, 417.12, 351.09, 276.43	Isoflavonoids	GG
67	9.856	C_26_H_28_O_13_	Puerarin 6 ^″^‐O‐xyloside	[M + H]^+^	549.1602	417.12, 399.11, 297.08	Isoflavonoids	GG
68	10.065	C_26_H_28_O_15_	Adonivernith	[M + H]^+^	581.1580	581.16, 449.11, 431.09, 329.07, 299.06	Flavonoids	
69	10.391	C_10_H_10_O_4_	Isoferulic acid	[M + H‐H_2_O]^+^	177.0549	177.05, 163.04, 145.03, 117.03	Phenylpropanoids	DG
70	10.636	C_17_H_23_NO_3_	Atropine	[M + Na]^+^	312.1551	312.16, 181.05, 114.10	Alkaloids	
71	10.755	C_21_H_24_O_10_	Phloretin‐2 ^′^‐O‐glucoside	[M−H]^−^	435.1267	435.13, 274.08, 273.08, 167.03, 123.05	Flavonoids	DS
72	10.831	C_22_H_22_O_11_	Scoparin	[M−H]^−^	461.1093	576.14, 575.07, 462.11, 461.11, 341.02	Flavonoids	HJT
73	11.027	C_27_H_30_O_16_	Kaempferol‐3‐o‐diglucoside	[M + H]^+^	611.2563	611.26, 449.10, 288.06, 287.06	Flavonoids	HQ, DS
74	11.636	C_16_H_12_O_5_	Calycosin	[M + H]^+^	285.0762	270.05, 253.05, 225.05	Isoflavonoids	HQ
75	11.651	C_22_H_22_O_10_	Sissotrine	[M + HCOO]^−^	491.1187	491.12, 445.11, 284.06, 283.06, 268.04	Isoflavonoids	GG
76	11.816	C_10_H_10_O_4_	Ferulic acid	[M + H]^+^	195.0613	195.06, 177.06	Phenylpropanoids	DG, DS
77	12.010	C_13_H_14_N_2_O_3_	N‐acetyltryptophan	[M−H]^−^	245.0928	245.09, 203.08, 201.11, 116.04	/	/
78	12.230	C_20_H_20_O_11_	Irisxanthone	[M−H]^−^	435.1385	345.10, 315.09, 272.06	/	GG
79	12.339	C_34_H_28_O_22_	1,2,3,6‐tetragalloylglucose	[M−H]^−^	787.0993	617.08, 465.07, 313.06, 169.01, 125.02	/	/
80	12.343	C_22_H_18_O_10_	Epicatechin gallate	[M−H]^−^	441.0844	289.07, 245.08, 169.01, 125.02	Flavonoids	HJT
81	12.412	C_21_H_22_O_10_	6‐O‐Caffeoylarbutin	[M−H]^−^	433.1183	161.02, 161.09, 133.03, 109.03, 108.02	Phenylpropanoids	/
82	12.439	C_27_H_22_O_12_	Lithospermic acid	[M−H]^−^	537.1057	517.15, 493.12, 313.07, 295.06, 185.02	Phenylpropanoids	CWZ
83	12.462	C_19_H_26_O_12_	Sucrose 6‐benzoate	[M + Na]^+^	469.1104	470.13, 469.11, 307.06	Phenolic acids	/
84	12.987	C_26_H_28_O_14_	7‐(2‐Apiosylglucosyl)apigenin	[M + H]^+^	565.1453	271.06	Flavonoids	/
85	13.332	C_18_H_16_O_5_	Puerol B	[M + H]^+^	313.1027	295.09, 267.10, 253.08, 107.05	Phenylpropanoids	GG
86	13.831	C_12_H_22_O_6_	9‐(2,3‐dihydroxypropoxy)‐9‐oxononanoic acid	[M−H]^−^	261.1341	187.10, 125.10	Fatty acids	/
87	14.120	C_21_H_20_O_11_	Astragaline	[M−H]^−^	447.0943	449.19, 448.10, 447.09, 285.03, 284.03	Flavonoids	HQ
88	14.182	C_41_H_32_O_26_	Pentagalloylglucose	[M−H]^−^	939.0974	939.10, 769.08, 617.06, 447.05, 169.02	Saccharides	CWZ
89	14.339	C_21_H_20_O_11_	Luteolin 7‐galactoside	[M−H]^−^	447.0925	284.03, 285.04, 227.06, 151.01	Flavonoids	DS
90	14.387	C_15_H_10_O_5_	7,3 ^′^,4 ^′^‐Trihydroxyflavone	[M + H]^+^	271.0606	197.93, 141.07, 137.02	Flavonoids	/
91	14.553	C_9_H_16_O_4_	Azelaic acid	[M−H]^−^	187.0936	187.10, 169.09, 124.09, 97.06	Fatty acids	DG
92	14.906	C_18_H_16_O_8_	Rosmarinic acid	[M−H]^−^	359.0773	359.08, 197.04, 179.03, 161.02, 133.03	Phenylpropanoids	DS
93	15.051	C_19_H_34_O_10_	(2R,3S,4S,5R,6R)‐2‐[[(2R,3R,4R)‐3,4‐dihydroxy‐4‐(hydroxymethyl)oxolan‐2‐yl]oxymethyl]‐6‐oct‐1‐en‐3‐yloxyoxane‐3,4,5‐triol	[M + HCOO]^−^	467.2033	421.21, 289.17, 161.04, 125.03, 101.02	/	/
94	15.108	C_23_H_22_O_10_	6 ^″^‐O‐acetyldaidzin	[M + H]^+^	459.1210	255.07	/	GG
95	15.141	C_27_H_30_O_15_	Luteolin‐7‐O‐rutinoside	[M−H]^−^	593.1509	285.04, 284.03	Flavonoids	GG
96	15.595	C_21_H_30_O_12_	3,4‐Dihydroxyallylbenzene 3,4‐di‐O‐glucoside	[M + Na]^+^	497.1419	497.14, 355.09	Phenylpropanoids	/
97	15.923	C_27_H_31_O_16_^+^	Cyanin	[M]^+^	611.1566	611.16, 450.11, 449.13, 287.00	/	DS
98	15.973	C_22_H_22_O_9_	Formononetin‐7‐O‐glucoside	[M + HCOO]^−^	475.1249	269.08, 268.07, 267.07, 253.05, 252.04	Isoflavonoids	HQ, GG
99	16.009	C_27_H_30_O_16_	Rutin	[M + H]+	611.1700	612.34, 611.17, 465.10, 303.05	Flavonoids	CWZ
100	16.146	C_36_H_30_O_16_	Salvianolic acid B	[M−H]^−^	717.1472	717.15, 519.09, 339.05, 321.04, 295.06	Phenylpropanoids	DS
101	16.418	C_15_H_10_O_4_	Daidzein	[M + H]^+^	255.0663	255.07, 199.08, 181.07, 137.02, 91.06	Isoflavonoids	GG
102	16.544	C_27_H_30_O_15_	Kaempferol 3‐O‐beta‐D‐glucopyranosyl‐7‐O‐alpha‐L‐rhamnopyranoside	[M + H]^+^	595.2445	288.06, 287.06, 287.03, 97.03, 85.03	Flavonoids	HQ, HJT
103	16.571	C_22_H_22_O_9_	Ononin	[M + H]^+^	431.1353	431.14, 269.08	Isoflavonoids	HQ, GG
104	16.718	C_23_H_24_O_10_	Wistin	[M + HCOO]^−^	505.1999	299.09, 298.08, 297.08, 283.06, 82.05	Isoflavonoids	/
105	17.015	C_21_H_20_O_12_	Spiraeoside	[M−H]^−^	463.1638	464.17, 463.15, 302.11, 301.11	Flavonoids	/
106	17.016	C_17_H_18_O_5_	Isomucronulatol	[M + H]^+^	303.0451	167.07, 161.06, 133.07, 123.05, 118.04	Isoflavonoids	HQ
107	17.024	C_21_H_20_O_10_	3,5‐dihydroxy‐2‐(4‐hydroxyphenyl)‐7‐[(3,4,5‐trihydroxy‐6‐methyloxan‐2‐yl)oxy]‐4H‐chromen‐4‐one	[M−H]^−^	431.0986	431.10, 285.05, 284.03, 257.04, 151.00	Flavonoids	/
108	17.038	C_21_H_20_O_10_	Afzelin	[M + H]^+^	433.1357	287.06	Flavonoids	HQ
109	17.152	C_19_H_36_O_10_	(2R,3R,4S,5S,6R)‐2‐octoxy‐6‐[[(2S,3R,4S,5R)‐3,4,5‐trihydroxyoxan‐2‐yl]oxymethyl]oxane‐3,4,5‐triol	[M + FA‐H]^−^	469.2305	423.22, 291.18	/	/
110	17.218	C_21_H_36_O_10_	(R)‐Linalyl beta‐vicianoside	[M + Na]^+^	471.2199	472.22, 471.22, 335.10, 334.55, 333.08	/	/
111	17.296	C_48_H_78_O_20_	Asiaticoside A	[M + HCOO]^−^	1019.509	1019.51, 974.50, 973.50, 469.23, 503.07	Triterpenoids	/
112	17.479	C_48_H_78_O_19_	Soyasaponin V	[M + H]^+^	959.5014	959.72, 599.40, 442.39, 441.38, 423.36	Triterpenoids	HQ
113	17.640	C_48_H_82_O_18_	Panax saponin C	[M + HCOO]^−^	991.4990	991.50, 947.54, 946.53, 945.53, 783.45	Triterpenoids	/
114	17.692	C_48_H_76_O_20_	(3beta,5xi,9xi,22beta)‐22,24,29‐Trihydroxy‐29‐oxoolean‐12‐en‐3‐yl 6‐deoxy‐alpha‐L‐mannopyranosyl‐(1‐>2)‐beta‐D‐galactopyranosyl‐(1‐>2)‐beta‐D‐glucopyranosiduronic acid	[M + FA‐H]^−^	1017.22	972.49, 971.49, 969.50, 645.35	Triterpenoids	/
115	17.801	C_15_H_10_O_5_	Genistein	[M−H]^−^	269.0447	271.05, 270.05, 269.04	Isoflavonoids	HQ, GG
116	18.067	C_48_H_78_O_19_	(S)‐asiaticoside	[M + HCOO]^−^	1003.5063	1003.51, 959.52, 958.51, 957.5, 469.12	Triterpenoids	/
117	18.444	C_15_H_12_O_4_	Isoliquiritigenin	[M−H]^−^	255.0672	255.07, 135.01, 120.06, 119.05, 91.02	Flavonoids	HQ, GG
118	18.513	C_16_H_10_O_5_	Pseudobaptigenin	[M−H]^−^	281.0445	282.05, 281.04, 254.06, 253.05, 252.04	Isoflavonoids	/
119	18.567	C_42_H_72_O_13_	Ginsenoside Rg3	[M + HCOO]^−^	829.4587	829.46, 785.46, 784.46, 783.48, 161.05	Triterpenoids	/
120	18.599	C_13_H_14_N_2_O_2_	Tetrahydroharman‐3‐carboxylic acid	[M + H]^+^	231.1006	231.12, 214.09, 188.08	Alkaloids	/
121	18.952	C_48_H_78_O_18_	Soyasaponin Bb	[M + HCOO]^−^	987.6950	987.696, 944.54, 943.52, 942.52, 941.51	Triterpenoids	HQ
122	18.974	C_48_H_76_O_19_	(3beta,5xi,9xi,22beta)‐22,24‐Dihydroxyolean‐12‐en‐3‐yl 6‐deoxy‐alpha‐L‐mannopyranosyl‐(1‐>2)‐beta‐D‐galactopyranuronosyl‐(1‐)‐beta‐D‐glucopyranosiduronic acid	[M + H]^+^	957.4994	423.36, 441.37, 353.07, 141.02, 85.03	Triterpenoids	HQ
123	18.997	C_48_H_78_O_18_	3‐Rha(1–2)Gal(1–2)GluA‐Soyasaponenol B	[M + FA‐H]^−^	987.5181	943.52, 942.52, 941.51, 247.08, 163.07	Triterpenoids	HQ
124	19.227	C_48_H_78_O_17_	[16beta,28‐Dihydroxy‐11,12,13,18‐tetradehydrooleanan‐3beta‐yl]4‐O‐(6‐deoxy‐alpha‐L‐mannopyranosyl)‐6‐O‐(beta‐D‐glucopyranosyl)‐beta‐D‐glucopyranoside	[M + HCOO]^−^	971.4941	971.49, 927.51, 926.52, 925.52, 779.46	Triterpenoids	HQ, DG
125	19.241	C_47_H_76_O_17_	(2S,3S,4S,5R,6R)‐6‐[[(3S,4S,4aR,6aR,6bS,8aR,9S,12aS,14aR,14bR)‐9‐hydroxy‐4‐(hydroxymethyl)‐4,6a,6b,8a,11,11,14b‐heptamethyl‐1,2,3,4a,5,6,7,8,9,10,12,12a,14,14a‐tetradecahydropicen‐3‐yl]oxy]‐5‐[(2S,3R,4S,5S)‐4,5‐dihydroxy‐3‐[(2S,3R,4R,5R,6S)‐3,4,5‐trihydroxy‐6‐methyloxan‐2‐yl]oxyoxan‐2‐yl]oxy‐3,4‐dihydroxyoxane‐2‐carboxylic acid	[M + HCOO]^−^	957.509	914.51, 913.55, 913.50, 912.50, 911.50	Triterpenoids	/
126	19.274	C_47_H_76_O_17_	(3beta,5xi,9xi,22beta)‐22,24‐Dihydroxyolean‐12‐en‐3‐yl 6‐deoxy‐alpha‐L‐mannopyranosyl‐(1‐>2)‐alpha‐L‐arabinopyranosyl‐(1‐>2)‐beta‐D‐glucopyranosiduronic acid	[M + H]^+^	913.1563	442.87, 441.37, 423.36, 203.18, 141.02	Triterpenoids	/
127	19.426	C_16_H_22_O_4_	Dibutyl phthalate	[M + H]^+^	279.1539	151.04, 149.02	Phenolic acids	DG
128	19.691	C_20_H_18_O_4_	Neobavaisoflavone	[M + H]^+^	323.1259	323.13, 323.05, 268.06, 267.06, 266.14	Isoflavonoids	GG
129	19.858	C_21_H_38_N^+^	Benzododecinium	[M]^+^	304.3015	304.30, 212.24, 91.06	/	/
130	20.130	C_47_H_83_O_13_P	[1‐[hydroxy‐(2,3,4,5,6‐pentahydroxycyclohexyl)oxyphosphoryl]oxy‐3‐octadecanoyloxypropan‐2‐yl] (8Z,11Z,14Z,17Z)‐icosa‐8,11,14,17‐tetraenoate	[M−H]^−^	885.5353	885.54, 581.32, 419.26, 303.24, 283.27	/	/
131	20.380	C_18_H_34_O_4_	12,13‐DiHOME	[M−H]^−^	313.2376	295.23, 183.14, 129.09	/	/
132	20.380	C_18_H_34_O_4_	9,10‐DiHOME	[M−H]^−^	313.2376	314.68, 314.13, 313.24, 297.11, 201.10	/	/
133	20.412	C_15_H_22_O_5_	Octyl gallate	[M−H]^−^	281.1385	281.14, 169.01, 168.00, 126.03, 124.02	Phenolic acids	DS
134	20.797	C_12_H_14_O_2_	3‐Butylidene‐1,3,4,5‐tetrahydro‐2‐benzofuran‐1‐one	[M + H]^+^	191.1063	191.11, 173.10, 145.11, 117.07, 91.05	/	/
135	21.660	C_19_H_20_O_3_	Cryptotanshinone	[M + H]^+^	297.1493	279.14, 251.14	/	DS
136	22.138	C_39_H_79_N_2_O_6_P	N‐palmitoylsphingomyelin	[M + HCOO]^−^	747.5466	690.55, 689.56, 688.55, 687.55, 227.19	/	/
137	22.504	C_19_H_18_O_3_	Tanshinone IIA	[M + H]^+^	295.1338	295.21, 295.19, 294.90, 294.99	/	DS
138	22.787	C_42_H_82_NO_8_P	1‐Palmitoyl‐2‐oleoyl‐sn‐glycero‐3‐phosphocholine	[M + Na]^+^	782.569	599.49, 577.51, 146.98, 86.10, 81.08	/	/
139	23.019	C_55_H_74_N_4_O_5_	Pheophytin a	[M + H]^+^	871.5762	594.28, 593.28, 592.27, 533.25, 460.2	/	/
140	23.448	C_23_H_46_N_6_O_13_	4,6‐Diamino‐2‐{[3‐o‐(2,6‐diamino‐2,6‐dideoxyhexopyranosyl)pentofuranosyl]oxy}‐3‐hydroxycyclohexyl 2,6‐diamino‐2,6‐dideoxyhexopyranoside	[M + Na]^+^	637.3057	637.31, 581.24, 525.18, 337.02, 147.12,	/	/
141	23.819	C_24_H_38_O_4_	Dioctyl phthalate	[M + H]^+^	391.2761	391.28, 262.17, 150.03, 149.02	Phenolic acids	/
142	23.911	C_22_H_43_NO	Erucamide	[2 M + H]^+^	675.6696	338.34	Fatty acids	/

**Figure 3:**
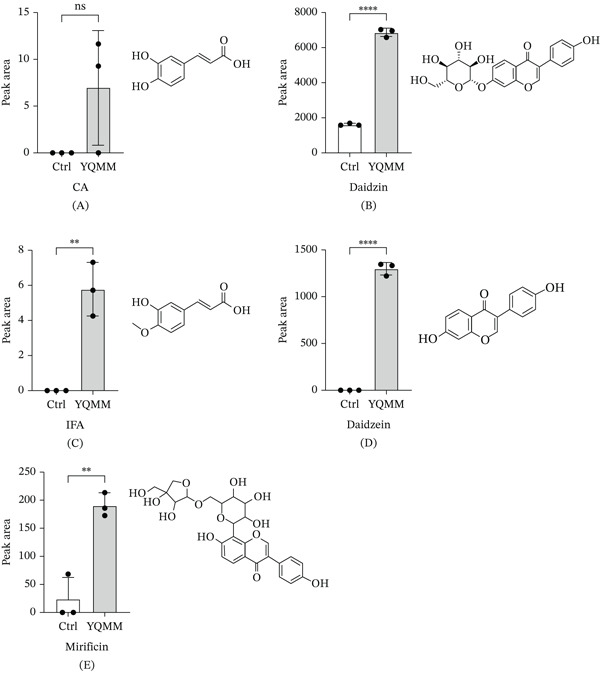
The compounds with prototype structures in plasma after YQMM treatment using the LC‐Q‐TOF/MS system. (A–E) Structural formulas of prototype compounds and peak areas of the compound in control group plasma versus treated group plasma.  ^∗∗∗∗^ *p* < 0.0001,  ^∗∗∗^ *p* < 0.001,  ^∗∗^ *p* < 0.01,  ^∗^ *p* < 0.05; ns: no significant difference. CA: caffeic acid; IFA: isoferulic acid.

Although the YQMM extract contains 142 chemical constituents, only those that can be absorbed into the bloodstream, namely the parent compounds, are most likely to exert direct pharmacological effects. In order to predict network pharmacology and validate in vitro activity, we gave priority to the prototype components found in mouse plasma after YQMM administration using LC‐Q‐TOF/MS as representative active pharmaceutical ingredients of YQMM. Five prototype compounds were detected in plasma and were also confirmed in the YQMM extract (Table 4). These included the isoflavonoids daidzin, daidzein, and mirificin, as well as the phenylpropanoids CA and daidzein were absent from the plasma of control mice but appeared after YQMM administration, as shown in Figure 3, but they emerged when YQMM was administered. Although baseline levels of daidzin and mirificin were present in control animals, their plasma concentrations were significantly modulated after treatment. The discovery of these absorbed prototype compounds offers a vital material basis for more research into the pharmacological processes and bioactive components of YQMM.

**Table 4:** Absorbed prototype compounds in mice plasma.

NO	RT(min)	Formula	Name	Adduct	MS	MS/MS	Category	Source
R4	3.813	C_9_H_8_O_4_	Caffeic Acid	[M−H]^−^	179.0321	179.03, 77.57	Phenylpropanoids	HQ, DS, HJT
R7	9.089	C_21_H_20_O_9_	Daidzin—20 eV	[M−H]^−^	415.1023	416.11, 415.10, 254.05, 253.05, 252.04	Isoflavonoids	GG
R8	9.905	C_26_H_28_O_13_	Mirificin	[M + H]^+^	549.1683	549.17, 417.13, 351.09	Isoflavonoids	GG
R9	10.317	C_10_H_10_O_4_	Isoferulic acid	[M + H‐H2O]^+^	177.0543	177.05, 145.03, 117.04	Phenylpropanoids	DG
R11	16.437	C_15_H_10_O_4_	Daidzein	[M + H]^+^	255.0657	255.07, 199.08, 181.06, 137.02	Isoflavonoids	GG

### 3.3. Network Pharmacology

#### 3.3.1. Target Prediction

**Figure 4:**
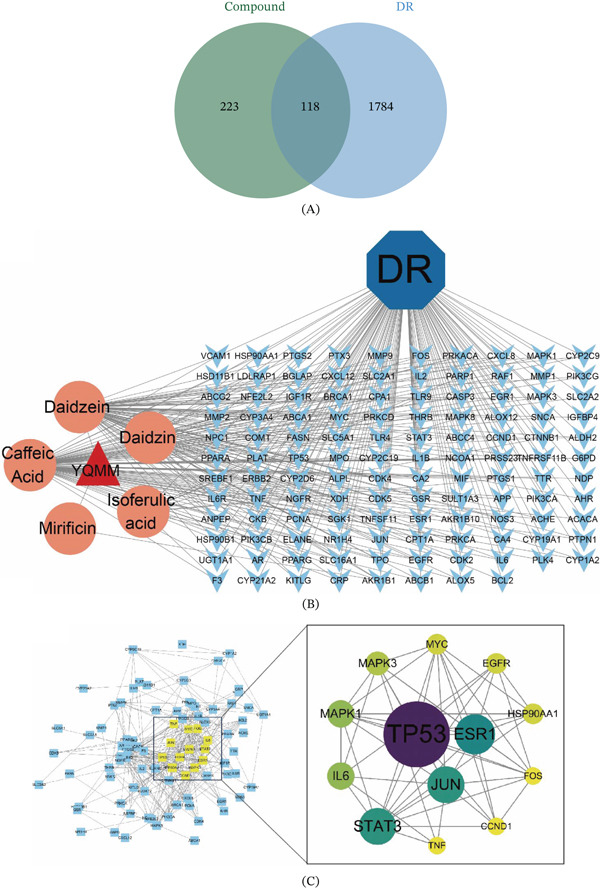
Network pharmacology study of YQMM for the treatment of diabetic retinopathy. (A) Venn diagram showing overlapping targets of YQMM prototype compounds derived from the PubChem database and SwissTargetPrediction target prediction database, alongside DR targets sourced from GeneCards, TTD, OMIM, and Drugbank databases. (B) Prototype compounds and 118 therapeutic targets are connected through compound‐target networks. Orange circles: active components of YQMM; light blue diamonds: intersection target. (C) The CytoNCA plugin screens the network, selecting nodes with betweenness, closeness, and degree values all exceeding the median as key hubs.

To elucidate the mechanisms by which YQMM exerts its effects, a network pharmacology strategy was adopted to systematically explore its potential biological targets and signaling pathways. Putative targets of the absorbed prototype compounds were retrieved from the SwissTargetPrediction and PubChem databases, yielding 341 unique targets after deduplication and standardization via UniProt. At the same time, 1902 DR‐related targets were gathered from illness databases such as DrugBank, OMIM, GeneCards, and TTD. The intersection of the two datasets yielded 118 overlapping targets (Figure 4A), which were identified as the potential therapeutic targets of YQMM against DR. To illustrate these relationships, a compound‐target‐disease network was built (Figure 4B).

To identify the core targets among these candidates, a PPI network was constructed using the STRING database. The topological characteristics of the network were further visualized via Cytoscape software (Figure 4C). The CytoNCA plugin was used for topological analysis. Key hubs were nodes whose degree, betweenness, and closeness values were all higher than the network median. In addition to the aforementioned parameters, this study further incorporated literature analysis. Priority was given to targets with well‐documented literature during DR pathology processes (e.g., TP53 regulates apoptosis [26]; STAT3, JUN, and ESR1 mediating inflammation and angiogenesis [27–29]; MAPK1 orchestrating cell proliferation and vascular formation [30]; IL6 is an inflammatory cytokine [31], as well as those located at intersections of multiple enriched pathways (e.g., STAT3 is present in both the AGE‐RAGE and PI3K‐Akt pathways) as core validation targets. These targets play critical roles in angiogenesis, cell proliferation, and inflammatory processes, all of which are essential components of the DR process.

#### 3.3.2. GO and KEGG Analysis

**Figure 5:**
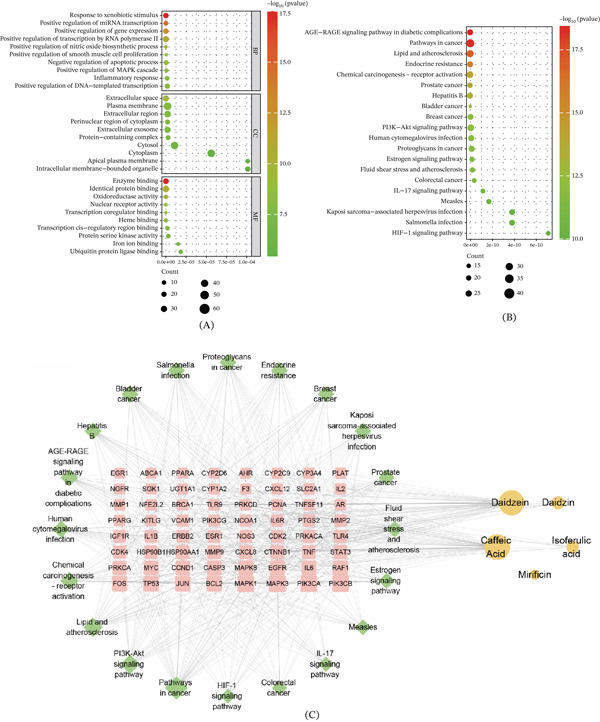
Results of GO and KEGG pathway enrichment analysis. (A) Top 10 BP, MF, and CC from GO analysis. (B) Top 20 KEGG enrichment analyses. (C) “Component‐Target‐Pathway” network. Yellow nodes represent components, pink nodes represent targets, and green nodes represent pathways.

Functional enrichment analysis of the 118 overlapping targets was performed using the DAVID platform. The enrichment analysis revealed a total of 792 GO terms. In terms of functional classification, biological processes (BP) accounted for the majority with 537 terms, followed by 76 cellular component (CC) terms and 179 molecular function (MF) terms. The Top 10 enriched GO terms in each category are visualized in Figure 5A. Among the BP, terms related to inflammatory signaling, upregulation of miRNA expression, activation of the MAPK cascade, and inhibition of apoptosis were prominent. At the CC level, enrichment was observed for terms associated with extracellular vesicles, plasma membranes, and apical plasma membranes. For MF, binding activities (enzyme binding, heme binding, and iron ion binding) and oxidoreductase activity were the most frequently enriched features.

Pathway analysis based on KEGG annotation further demonstrated that these shared targets clustered in pathways implicated in the pathophysiology of DR. Including AGE‐RAGE signaling, PI3K‐Akt signaling, and HIF‐1 signaling (Figure 5B). These findings are consistent with previous reports linking these networks to DR development [32–34], suggesting their involvement in YQMM′s mechanism of action.

To further integrate this information, a new network was constructed (Figure 5C), illustrating the complex interplay between active ingredients, molecular targets, and biological pathways. This network highlights that individual compounds can act on multiple targets and that distinct targets can converge on the same pathway, reflecting a coordinated, multilevel mechanism through which YQMM may exert therapeutic effects in DR.

### 3.4. Molecular Docking

**Figure 6:**
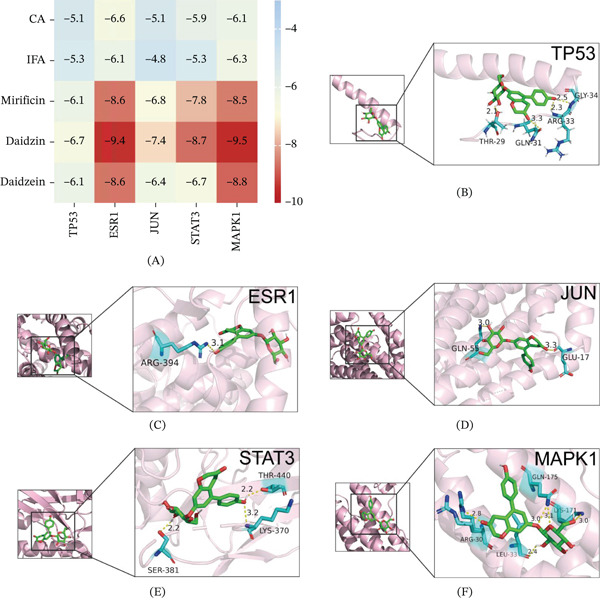
YQMM prototype compounds exhibit strong binding activity and docking with key target molecules. (A) Heatmap of binding energies from docking results between compounds and target protein molecules. (B–F) Visualization of docking results between daidzin and target molecules.

Molecular docking simulations were used to predict the binding affinities between the absorbed prototype compounds in YQMM and the core targets identified by network pharmacology, with binding energy used to evaluate the strength of compound‐target interactions. The results show (Figure 6A) that all five components in YQMM exhibited favorable binding potential with key target proteins, with binding energies consistently below −4.9 kcal/mol. Binding energy analysis revealed that soybean glycosides demonstrated the highest affinity for each target protein, forming the most stable complex conformation, which was visually illustrated (Figure 6B–F).

### 3.5. Experimental Validation of Predicted Core Targets

**Figure 7:**
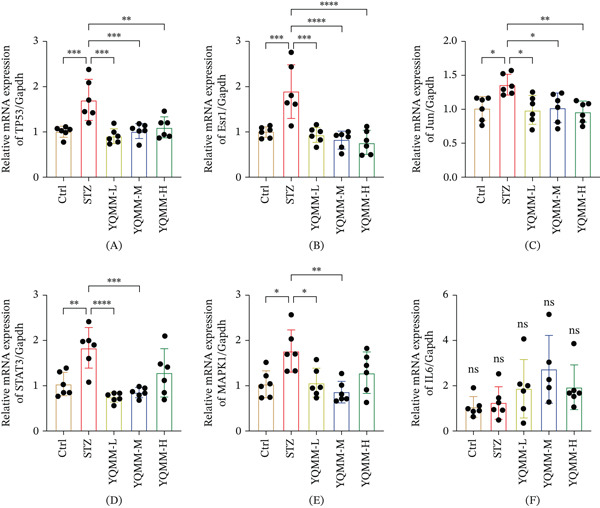
(A–F The regulatory effects of YQMM on key targets were validated by RT‐qPCR. The mRNA expression levels of TP53, ESR1, JUN, STAT3, IL6, and MAPK1 in mice retinal tissues were determined by qPCR. Data are expressed as mean ± SD (*n* = 6). ^∗∗∗∗^ *p* < 0.0001,  ^∗∗∗^ *p* < 0.001,  ^∗∗^ *p* < 0.01,  ^∗^ *p* < 0.05; ns: no significant difference.

Quantitative RT‐PCR was performed on retinal specimens from the DR model to quantify transcripts corresponding to the computationally shortlisted targets, thereby assessing whether YQMM alters these disease‐related molecular readouts. Findings are displayed in Figure 7. In our study, YQMM treatment downregulated the expression of TP53, ESR1, JUN, STAT3, and MAPK1, consistent with previously reported findings [35–38]. Nevertheless, our findings showed that YQMM therapy had no discernible regulatory impact on IL6. According to these findings, YQMM enhances DR by controlling TP53, ESR1, JUN, STAT3, and MAPK1.

### 3.6. YQMM Inhibits the Proliferation and Tube Formation of HUVECs In Vitro

**Figure 8:**
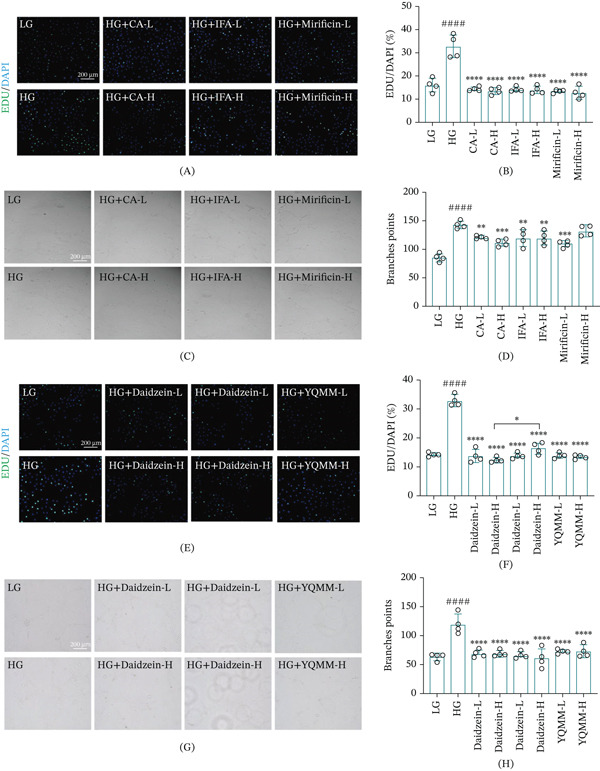
Drug active ingredients and YQMM improve HG‐induced dysfunction in HUVECs. (A–B) Representative images of EDU staining for CA, IFA, and (A) mirificin and (B) quantitative analysis, with cell nuclei stained by DAPI (blue); (C–D) Tube formation assay and branching points in HUVEC cells. (E–F) Representative images and quantitative analysis of EDU staining for daidzin, daidzein and YQMM; nuclei stained with DAPI (blue). (G–H) Tube formation assay and branching points in HUVECs (magnification: 10×; scale bar = 200 *μ*m.). Data are presented as mean ± standard deviation (*n* = 4). #: Compared with LG, ^####^ *p* < 0.0001, ^###^ *p* < 0.001, ^##^ *p* < 0.01, ^#^ *p* < 0.05;  ^∗^: Compared with HG,  ^∗∗∗∗^ *p* < 0.0001,  ^∗∗∗^ *p* < 0.001,  ^∗∗^ *p* < 0.01,  ^∗^ *p* < 0.05. LG: 5.5 mM D‐glucose; HG: 30 mM D‐glucose; CA‐L: 1 *μ*M; CA‐H: 10 *μ*M; IFA‐L: 5 *μ*M; IFA‐H: 50 *μ*M; mirificin‐L: 10 *μ*M; mirificin‐H: 50 *μ*M; daidzin‐L: 5 *μ*M; daidzin‐H: 50 *μ*M; daidzein‐L: 5 *μ*M; daidzein‐H: 10 *μ*M; YQMM‐L: 1 *μ*g/mL; and YQMM‐L: 10 *μ*g/mL.

The effects of YQMM and its absorbed prototype compounds on HG‐induced endothelial dysfunction were assessed in vitro. First, a CCK‐8 experiment verified that a 24 h exposure to 30 mM glucose greatly increased HUVECs proliferation (Figure S1A). Cytotoxicity screening revealed that whereas mirificin and daidzein exhibited cytotoxic or pro‐proliferative effects at 100 *μ*M and 20 *μ*M, respectively, CA, IFA, daidzin, and YQMM extract did not impact cell viability within the investigated dose ranges (Figure S1B–G). These findings led to the selection of noncytotoxic doses for the following functional tests: YQMM extract (1 *μ*g/mL and 10 *μ*g/mL), CA (1 *μ*M and 10 *μ*M), IFA (5 *μ*M and 50 *μ*M), mirificin (10 *μ*M and 50 *μ*M), daidzin (5 *μ*M and 50 *μ*M), and daidzein (5 *μ*M and 10 *μ*M). EdU incorporation assays showed that HG stimulation markedly increased HUVEC proliferation, and this effect was significantly reduced by cotreatment with either the prototype compounds or the YQMM extract at both low and high concentrations (Figure 8A, B, E, and F). Simultaneously, tube formation experiments showed that HG improved HUVECs′ angiogenic potential, as shown by more branching points. All investigated drugs including YQMM extract successfully inhibited this proangiogenic response (Figure 8C, D, G, and H).

Collectively, the experimental data indicated that YQMM and its active compounds effectively suppressed HG‐induced endothelial cell proliferation and capillary‐like tube formation. These findings suggest that YQMM may alleviate DR‐associated pathological neovascularization.

### 3.7. In Vitro Target Validation

To investigate the molecular mechanism behind the antiangiogenic effects noted above, we investigated whether YQMM and its active compounds might control the expression of the anticipated targets in HUVECs. Because HUVECs lack ESR1 expression [39], we focused our investigation on the remaining four key targets: TP53, JUN, STAT3, and MAPK1.

**Figure 9:**
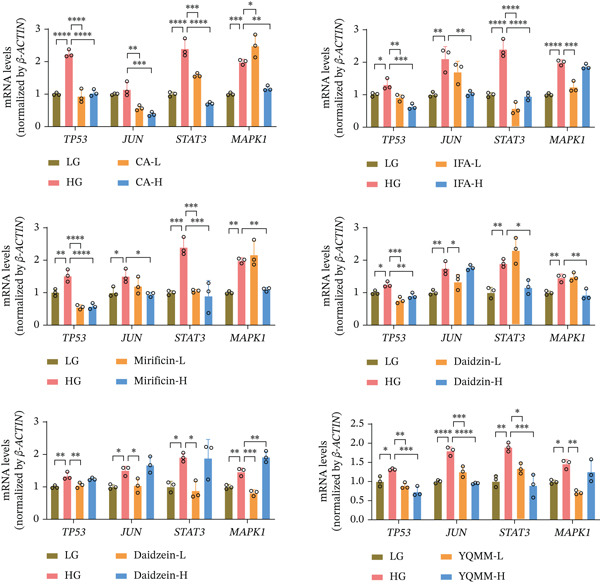
The regulatory effects of various active compounds and YQMM on key targets were validated via RT‐qPCR. RT‐qPCR was employed to detect mRNA expression levels of TP53, JUN, STAT3 and MAPK1 induced by high glucose. Data are presented as mean ± standard deviation (*n* = 3).  ^∗∗∗∗^ *p* < 0.0001,  ^∗∗∗^ *p* < 0.001,  ^∗∗^ *p* < 0.01,  ^∗^ *p* < 0.05. LG: 5.5 mM D‐glucose; HG: 30 mM D‐glucose; CA‐L: 1 *μ*M; CA‐H: 10 *μ*M; IFA‐L: 5 *μ*M; IFA‐H: 50 *μ*M; mirificin‐L: 10 *μ*M; mirificin‐H: 50 *μ*M; daidzin‐L: 5 *μ*M; daidzin‐H: 50 *μ*M; daidzein‐L: 5 *μ*M; daidzein‐H: 10 *μ*M; YQMM‐L: 1 *μ*g/mL; YQMM‐L: 10 *μ*g/mL.

When compared with the normal glucose control, high glucose stimulation strengthens the mRNA expression of TP53, JUN, STAT3, and MAPK1, according to qPCR analysis. These increases were considerably reduced to varied degrees by treatment with YQMM extract or each of the five prototype compounds (Figure 9). The downregulation of these key genes aligns with the observed inhibition of endothelial cell proliferation and tube formation.

These results indicate that the reduced expression of TP53, JUN, STAT3, and MAPK1 is associated with the protective effects of YQMM and its bioactive components against HG‐induced endothelial dysfunction. This pattern of multitarget regulation supports a synergistic mechanism through which YQMM may help prevent the progression of DR.

## 4. Discussion

DR represents a prevalent microvascular complication arising from diabetes, characterized by a complex interplay of pathological processes. Throughout the course of the disease, aberrant activation of inflammatory cascades, oxidative damage, neural tissue degeneration, and disruptions in vascular integrity contribute to progressive retinal injury [40–42]. Although current clinical interventions such as anti‐VEGF therapy and laser photocoagulation can delay disease progression to some extent, limitations remain, including high treatment costs, the need for repeated administration, and an inability to fully reverse visual impairment [4, 5].

Because of their favorable safety profiles, an increasing number of clinically effective herbal formulas are being used alongside conventional western therapies [43]. Available evidence suggests that herbal treatments can mitigate common diabetic complications while producing fewer side effects [44]. DR is classified as “Wasting‐Thirst Eye Disease” in the theoretical framework of TCM. Dual Qi and Yin deficiencies are central to its pathogenesis. In traditional theory, a “root deficiency with branch excess” in DR manifests as blood stasis obstructing the vessels—a key pathogenic factor that leads to retinal hemorrhages and microvascular damage [45, 46]. TCM often employs holistic treatment strategies—for example, nourishing Yin and strengthening the kidneys, invigorating Qi, promoting blood circulation to dispel stasis, and clearing heat to cool the blood—all guided by syndrome differentiation principles [47]. As DR develops through a cascade of interconnected pathological events, there is a critical need for interventions that can simultaneously target multiple molecular pathways and processes. TCM formulations, which contain diverse bioactive constituents, are well suited to address this complexity because of their inherent multitarget pharmacological profiles, thereby offering an alternative therapeutic strategy for DR management [48].

TCM herbal formulas are traditionally formulated according to the “Monarch, Minister, Assistant, and Guide” principle (Jun‐Chen‐Zuo‐Shi in Chinese). This hierarchical framework illustrates the synergistic interactions among herbs within a prescription, whereby monarch herbs target the primary disorder, minister herbs enhance efficacy, assistant herbs address secondary symptoms or reduce toxicity, and guide herbs direct or harmonize the overall therapeutic effect [49]. In the YQMM formulation, Huangqi and Danshen serve as the principal (“monarch”) herbs and have long been incorporated in therapies aimed at DR. Research has demonstrated that Huangqi possesses multiple pharmacological activities, including lowering blood glucose, alleviating inflammation, counteracting oxidative stress, and inhibiting abnormal vessel growth in the retina [13]. Among its bioactive constituents, astragaloside IV has been shown to influence the HIF‐1*α*/VEGF signaling axis, thereby reducing hypoxia driven neovascularization in retinal tissues [50]. Danshen exhibits notable neuroprotective, cardioprotective, anti‐inflammatory, and antioxidant properties [51]. Evidence from both cell‐based studies and animal models indicates that the bioactive constituents of YQMM significantly suppress abnormal retinal neovascularization by impairing endothelial cell migration, limiting proliferative responses, and disrupting capillary‐like network formation [52]. The minister herb Gegen prevents leukocyte adhesion to and damage of retinal capillaries by suppressing IL‐1*β*‐triggered inflammatory responses and cell apoptosis [14]. Salidroside—an active compound from Hongjing Tian—has demonstrated efficacy in ameliorating diabetes and its complications [53]. As guide herbs in the formula, Chongwei Zi and Danggui enhance microvascular circulation, relieve retinal hypoxia and ischemia, and curb abnormal angiogenesis in DR [10, 54].

The active ingredients and molecular mechanisms of YQMM, a traditional Chinese herbal remedy used in clinical settings to cure DR, are still unclear. By identifying YQMM′s nonglucose‐dependent protective effects, this study fills in knowledge gaps regarding the pharmacological basis and systematic mechanisms of action of the drug. This provides new information for the development of DR treatment strategies that do not depend on blood glucose control. A three‐tier pipeline—covering constituent profiling, in silico target/pathway mapping, and bench confirmation—was applied to elucidate how YQMM may alleviate DR. Initially, the active compounds and key components absorbed from YQMM were identified using LC‐Q‐TOF/MS, highlighting important bioactive chemicals such as CA, IFA, daidzin, daidzein, and mirificin. Subsequently, network pharmacology and molecular docking were performed to predict the key targets, including TP53, ESR1, JUN, STAT3, and MAPK1; as well as the signaling pathways closely associated with the pathogenesis of DR, such as the AGE‐RAGE, PI3K‐Akt, and HIF‐1 pathways. Experimental validation, both in vitro and in vivo, confirmed that YQMM and its active constituents significantly alleviated retinal vascular abnormalities in DR mice, while also inhibiting high glucose‐induced endothelial cell proliferation and tube formation in HUVECs.

The core targets identified in this study play crucial roles in the pathological process of DR. Notably, the Hexuemingmu Tablet (comprised of Huangqi, Danshen, Chongwei Zi, and Danggui) and the herb Gegen ameliorate DR by modulating targets like TP53, ESR1, and STAT3, mirroring our findings [54, 55]. High glucose exposure can activate the TP53 pathway, a tumor suppressor mechanism, leading to oxidative stress, cellular senescence, and death in retinal endothelial cells [35, 56]. Loss of estrogen receptor *α* function is strongly associated with metabolic disturbances such as impaired glucose tolerance and insulin resistance [36, 57], suggesting its potential involvement in early metabolic dysregulation in DR. Although ESR1 is not expressed in HUVECs, this may indicate limitations in network pharmacology predictions and in vivo/in vitro experimental results, while also highlighting the rigor of integrating in vivo, in vitro, and computational approaches. STAT3 and c‐JUN are pivotal transcription factors involved in multiple pathological pathways (including inflammation, cell proliferation, apoptosis, and angiogenesis) [37, 38].

A review of the literature indicates that daidzin inhibits the JAK2/STAT3 pathway and reduces IL6 secretion, thereby blocking the IL6/STAT3‐mediated proliferation and angiogenesis [58]. Molecular docking studies confirm that daidzin directly binds to the ESR1, inhibits JUN transcriptional activity, and the STAT3/MAPK1 signaling pathway, downregulates IL6‐related inflammatory factors, and thereby suppresses cell proliferation and angiogenesis [59]. Daidzin also inhibits MAPK1 (ERK1/2) phosphorylation, reduces IL6 and TNF‐*α* levels, and alleviates inflammation‐induced angiogenesis and matrix remodeling via the MAPK1/IL‐6 axis [60]. Daidzein blocks STAT3 phosphorylation and inhibits MAPK1 activation, downregulating the IL‐6/JUN and AP‐1/JUN signaling pathways; it reduces IL6 secretion, thereby inhibiting VEGF‐mediated cell proliferation, invasion, endothelial migration, and angiogenesis [61, 62]. Existing studies have demonstrated that, in cellular models exposed to high glucose, CA inhibits STAT3 and ERK1/2 phosphorylation as well as IL6 production; this confirms that the IL6/STAT3/MAPK1 pathway is the core mechanism underlying CA′s antiangiogenic effects in high‐glucose environments [63]. In addition, CA acts on ESR1 to inhibit c‐Jun/AP‐1 and other factors, thereby improving abnormal proliferation and angiogenesis [64, 65]. IFA effectively inhibits glucose‐mediated protein glycation and alleviates HG‐induced cellular damage. By regulating TP53‐related apoptosis and autophagy pathways, inhibiting Akt/mTOR signaling, and simultaneously modulating pathways such as MAPK/ERK and JNK/c‐Jun, this approach suppresses abnormal cell proliferation, thereby providing a potential mechanism for improving hyperglycemia‐associated vascular abnormalities [66–68]. Some studies have reported that mirificin inhibits abnormal cell proliferation [69].

These literature reports suggest that CA, IFA, daidzin, daidzein, and mirificin can downregulate the expression levels of these target genes, which is consistent with our findings. Therefore, we speculate that YQMM and its active ingredients may simultaneously regulate multiple mechanisms, including apoptosis, metabolic dysregulation, and inflammatory responses, through these targets, thereby suppressing pathological cell proliferation and angiogenesis.

Furthermore, network enrichment analysis suggested that pathways such as AGE‐RAGE, PI3K‐Akt, and HIF‐1 may represent pivotal hubs through which YQMM exerts its effects. Over‐activation of the AGE–RAGE signaling axis can worsen oxidative stress under diabetic conditions [32]; the PI3K‐Akt pathway is a central pathway regulating cell survival and metabolism [33, 70]; and HIF‐1*α* is a key driver of pathological retinal neovascularization [71, 72]. The intricate signaling network of DR is made up of these linked pathways. By acting at several nodes in this network, YQMM may have synergistic therapeutic benefits.

In summary, this study preliminarily clarified the potential pharmacodynamic substances of YQMM for treating DR and its multitarget mechanism of action centered on TP53, STAT3, JUN, among others, providing experimental evidence for its clinical application. Future research should use techniques like western blot, immunofluorescence, and pathway inhibitors in animal and cellular models to further validate hypothesized pathways like AGE‐RAGE and PI3K‐Akt. Additionally, clarifying the interactions among the various active components will be necessary for a more complete interpretation of the scientific basis of this formula.

## 5. Conclusion

**Figure 10:**
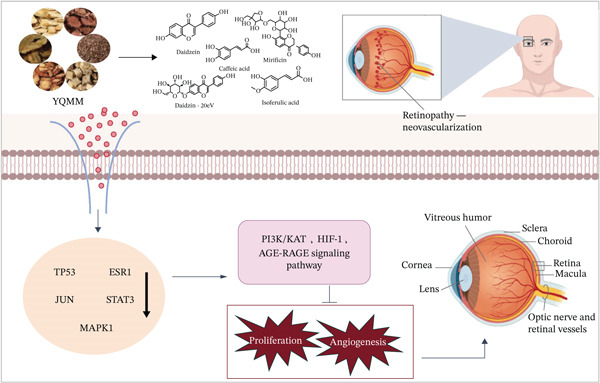
Schematic diagram of YQMM improving DR by regulating targets such as TP53, ESR1, JUN, and STAT3, as well as modulating key pathways including AGE‐RAGE, PI3K‐Akt, and HIF‐1.

In summary, research shows that YQMM exerted retinal protective effects, whereas no significant alterations in systemic blood glucose levels were observed. This phenomenon may be mediated by regulating core targets including TP53, ESR1, JUN, and STAT3, as well as modulating pivotal signaling pathways such as AGE‐RAGE, PI3K‐Akt, and HIF‐1 (Figure 10). Furthermore, in vitro experiments verified that YQMM and its active ingredients (CA, IFA, daidzein, daidzin, and mirificin) could effectively ameliorate HG‐induced endothelial dysfunction. Together, these findings validate the “multi‐component, multi‐target” pharmacological basis of YQMM and support its development as a potential therapeutic for DR. This work offers both theoretical insight and practical guidance for future drug discovery efforts targeting this complex disease. Future studies will focus on further validating the implicated pathways, exploring synergistic interactions among active compounds, and evaluating long‐term efficacy and safety to promote the clinical application of this TCM formula for DR prevention and treatment. Research avenues include combination optimization and formulation development, which have important ramifications for advancing the modernization and internationalization of TCM.

## Author Contributions

**Zhu-jun Mao:** writing – review and editing, methodology, data curation. **Qiang Lyu:** methodology, writing – review and editing. **Yuan Gao:** writing – original draft, visualization, validation, data curation. **Si-wei Wang:** validation. All authors discussed and commented on the manuscript. Yuan Gao and Qiang Lyu have contributed equally to this work.

## Funding

This work was supported by the Quzhou technology projects, China (2024K106).

## Conflicts of Interest

The authors declare no conflicts of interest.

## Supporting information


**Supporting Information** Additional supporting information can be found online in the Supporting Information section. Materials and methods: Construction of “Component‐Target‐Pathway” network. Figure S1: Effects of drugs on cell viability.

## Data Availability

The data that support the findings of this study are available from the corresponding author upon reasonable request.
