# Supplementary material for: An Integrated Approach Combining Chemical Profiling, Network Pharmacology, and Experimental Validation Is Used to Clarify the Pharmacological Basis of the Yiqi‐Tongluo‐Huoxue‐Mingmu Formula in Diabetic Retinopathy
Source: J Diabetes Res. 2026 May 28;2026:8484553. doi: 10.1155/jdr/8484553 (PMC13238508; doi:10.1155/jdr/8484553)

**An integrated approach combining chemical profiling, network pharmacology, and experimental validation is used to clarify the pharmacological basis of the Yiqi-Tongluo-Huoxue-Mingmu Formula in diabetic retinopathy**

Yuan Gao^a,1^ ,Qiang Lyu^a,1^ ,Si-wei Wang^b^ ,Zhu-jun Mao^a*^

^a^ School of Pharmaceutical Sciences, Zhejiang Chinese Medical University,Hangzhou 310053, China

^b^ Panvascular Diseases Research Center, the Quzhou Affiliated Hospital of Wenzhou Medical University, Quzhou People’s Hospital, Quzhou 324000, China

^1^ These authors contribute equally to this work.

Author’s e-mail: Yuan Gao, [gyuan0527@163.com](mailto:gyuan0527@163.com); Qiang Lyu, [lyuqiang@zju.edu.cn](mailto:lyuqiang@zju.edu.cn); Si-wei Wang, [wsw_1972@wmu.edu.cn](mailto:wsw_1972@wmu.edu.cn)

* Corresponding author at: School of Pharmaceutical Sciences, Zhejiang Chinese Medical University, No 548 Binwen Road, Hangzhou 310053, China.

E-mail addresses: [maozhujun0107@zcmu.edu.cn](mailto:maozhujun0107@zcmu.edu.cn).

**Materials and methods**

**Construction of “Component-Target-Pathway” network**

The “component-target-pathway” network by integrating the components, targets, and important pathways found by KEGG enrichment analysis of the YQMM formula using Cytoscape software (version 3.10.3).

**Figure legends**

Figure S1： Effects of drugs on cell viability. (A) CCK8 assay at different glucose concentrations. (B–G) CCK8 assay at different drug concentrations. Data are expressed as mean ± standard deviation (n = 6).

**Figure S1**


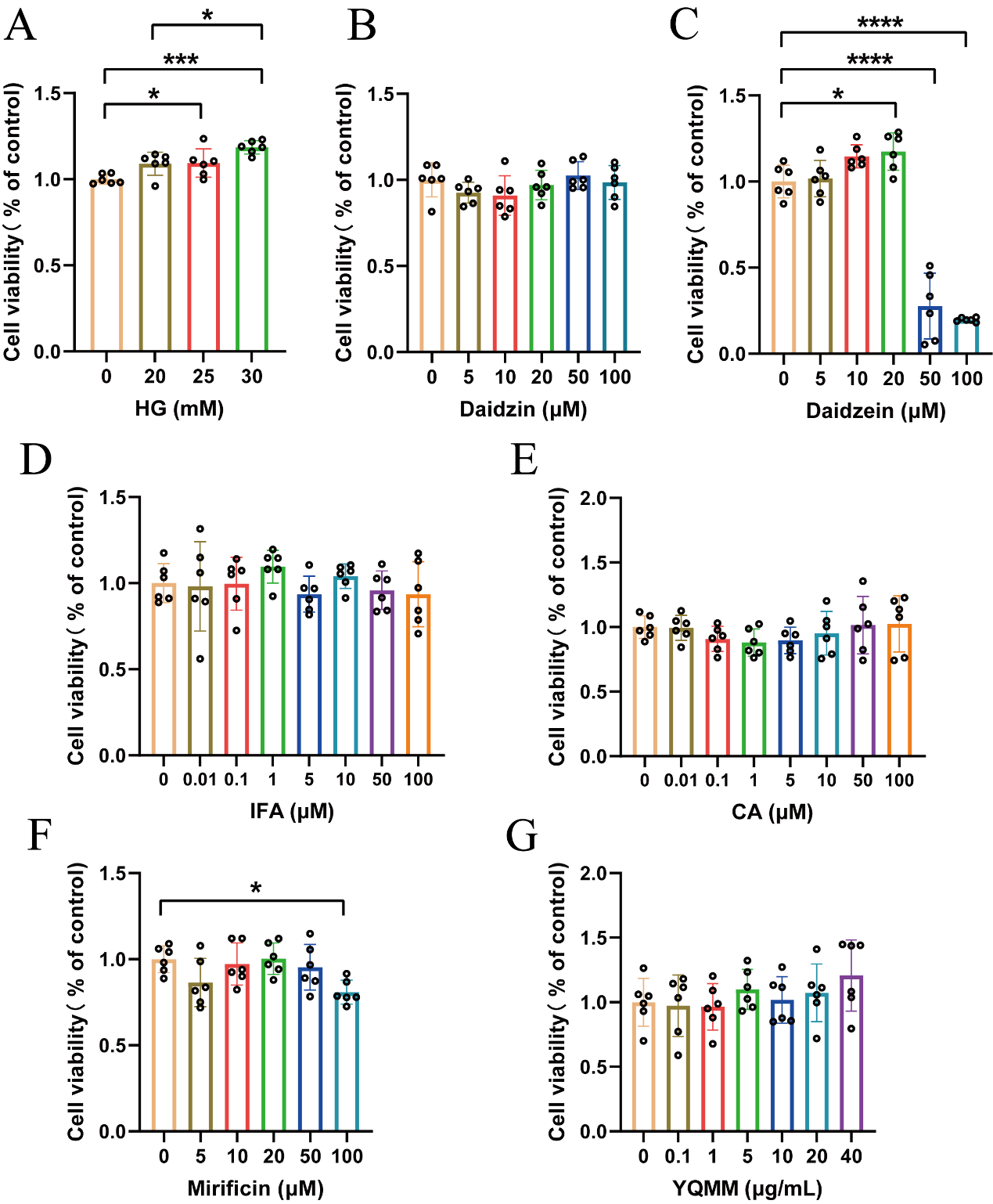

Supplement: Supplementary file 1 — Supporting Information Additional supporting information can be found online in the Supporting Information section. Materials and methods: Construction of “Component‐Target‐Pathway” network. Figure S1: Effects of drugs on cell viability. [file JDR-2026-8484553-s001.docx]
